# Perceptions of In‐Home Usage Experience and Price: Results of Consumer Research on Nutritional Innovations for Improving Maternal and Child Nutrition in Ethiopia

**DOI:** 10.1002/fsn3.4758

**Published:** 2025-02-28

**Authors:** Rebecca Olson, Puja Peyden Tshering, Kalpana Beesabathuni, Srujith Lingala, Afomiya Mekonnen, Masresha Tessema, Alemnesh Petros, Tarik Taye Birhanu, Abeba Ayele, Sufia Askari, Rowena K. Merritt

**Affiliations:** ^1^ Sight and Life Switzerland; ^2^ Ethiopian Public Health Institute Ethiopia; ^3^ Children's Investment Fund Foundation UK; ^4^ University of Kent Canterbury England

**Keywords:** eggs, Ethiopia, nutritional supplementation, product prototypes, qualitative study, user experience

## Abstract

Despite progress in improving maternal and child nutrition, there are still high levels of stunting and anemia in Ethiopia, primarily caused by dietary diversity and low consumption of animal‐sourced foods (ASFs). One promising solution is the utilization of egg powder, for it requires reduced transport and storage costs, has an extended shelf life, and versatile usage compared to whole eggs. A consumer research study conducted by Sight and Life (SAL) and the Ethiopian Public Health Institute (EPHI) aimed to explore opportunities for introducing powdered egg products targeting pregnant and lactating women (PLW) and children 6–60 months old in Ethiopia. The qualitative study assessed participants' usage of egg powder across four prototypes: (i) plain egg powder, (ii) Shiro with egg powder, (iii) porridge mixed with egg powder, and (iv) egg powder scrambled with the local flatbread (injera). The research focused on participants' reactions to each prototype, considering preparation, cooking, and consumption experience, and gauged perceptions regarding each prototype's relevance, pricing, packaging, and place of purchase. A total of 38 participants were recruited across four regions with the following criteria: (1) Mothers with at least one child under 5; (2) PLW; (3) Decision‐makers in their households regarding food purchases; (4) Literate, to ensure comprehension of the recipe booklet and label elements; (5) Not averse to the consumption of eggs; (6) No history of egg allergies (including family members); and (7) Belonging to lower‐income categories. Overall, the egg powder received positive feedback, with identified areas for improvement indicated, mostly from an organoleptic point of view (unpleasant smell during the preparation stage). An in‐home usage study of egg powder revealed positive reactions, particularly for the Shiro and baby porridge prototypes. The results are critical in identifying and introducing the most appropriate egg powder product for women and children in Ethiopia.

## Graphical abstract

Egg powder is a promising solution to address malnutrition that offers several benefits over whole eggs such as reduced transport/storage costs, increased shelf‐life, and offers a wide range of uses. A consumer research study was conducted by Sight and Life (SAL) in collaboration with the Ethiopian Public Health Institute (EPHI) to understand and frame opportunities‐for‐entry for a powdered egg product targeting pregnant and lactating women (PLW) and children 6–60 months old in Ethiopia. The study found that the egg powder prototypes were met with positive reactions, with clear areas of improvement indicated.

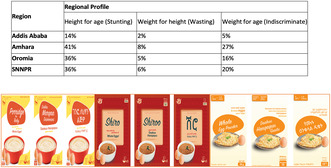

## 1 Introduction

Ethiopia has made significant strides in improving nutrition and health outcomes over the past two decades, yet malnutrition rates remain high, and progress has stagnated in certain areas. Between the years 2000 and 2016, the rate of stunting declined from 57.7% to 38.4%; however, over one‐third of children below the age of five are still affected, with considerable regional variation which ranges from 46.3% in Amhara to 14.5% in Addis Ababa (CSA and ICF 2016). Even among the wealthiest households, a quarter of children are stunted (CSA and ICF 2016). Additionally, 56.9% of children under five suffer from anemia. Poor complementary feeding practices contribute to these high malnutrition rates, with only 12.5% of children between six and 23 months meeting the minimum dietary diversity score and only 7% receiving a minimally acceptable diet (Kumera, Tsedal, and Ayana 2018). Many children are introduced to complementary foods too early or too late, with only 60% being introduced to semi‐solid and solid foods at the recommended 6–8 months (Zerfu, Umeta, and Baye 2016). The majority of complementary foods consist of grains, pulses, and vegetables, and only 17% of children aged six to 23 months consume eggs (CSA and ICF 2016).

Maternal nutrition is also a cause for concern, with 23.6% of women classified as anemic (CSA and ICF 2016) and pregnant and lactating women (PLW) generally have very poor dietary diversity (Zerfu, Umeta, and Baye 2016; Nana and Zema 2018). Over 10% of all babies born in Ethiopia are of low birth weight (< 2500 g at term), increasing their risk of death and impaired growth and development (Kumera, Tsedal, and Ayana 2018). Malnutrition among women results from poor‐quality diets, inadequate nutrition services, and suboptimal caring practices. Women's diets are typically deficient in essential nutrition due to limited intake of animal products, fruits, and vegetables. These deficiencies are exacerbated during pregnancy due to increased physiological needs, leading to poor birth outcomes and higher maternal mortality (Keats et al. 2021; Darnton‐Hill and Mkparu 2015).

Eggs are a highly nutritious food that provides a wide array of essential nutrients, making them particularly beneficial for the health and development of children. They are an excellent source of high‐quality protein and essential vitamins and minerals like choline, which supports cognitive development‐ and vitamin D, which supports bone health by enhancing calcium absorption (Zeisel 2011; Dawson‐Hughes 2008). The nutrient bioavailability in eggs is notably high, allowing for effective absorption and utilization by the body, thus making eggs an efficient and valuable component of a child's diet (Micha et al. 2017).

Global evidence shows that egg consumption positively affects children's growth and reduces stunting rates (Iannotti et al. 2017). However, in Ethiopia, the consumption of animal‐source foods (ASFs) remains low, particularly among children and PLW. Eggs are rich in essential nutrients such as vitamins A B, D, and E, vitamin selenium, choline, iron, iodine, calcium, phosphorus, potassium, and zinc, and provide numerous health benefits (Lutter, Iannotti, and Stewart 2018). For pregnant women, two 50‐g eggs provide 18% of the recommended daily allowance (RDA) for protein, provide 20%–35% of the RDA for vitamin A, riboflavin, pantothenic acid, vitamin B12, and phosphorus, and over 50% of RDA for choline and selenium. The same number of eggs for lactating women provides between 20% and 35% of the requirements for riboflavin, pantothenic acid, vitamin B12, iron, and phosphorus (Iannotti et al. 2018).

The Ethiopian diet predominantly consists of cereals, grains and vegetables, with limited consumption of ASFs (Abegaz, Hassen, and Minten 2018; Kumera, Tsedal, and Ayana 2018). Among ASFs, milk and dairy products are consumed more frequently than meat and eggs (Hafebo et al. 2015; Workicho et al. 2016; Abegaz, Hassen, and Minten 2018). Although Ethiopians recognize eggs as nutritious food, their consumption is hindered by factors such as price, availability and religious practices. When eggs are included in a meal, they are added to the common family pot (Mekonnen 2014) leaving the most in need with an amount less than they should consume.

Despite ongoing nutrition education efforts, dietary diversity in Ethiopia remains low, and egg consumption is suboptimal due to high costs and limited availability (Hailemichael et al. 2016; Hirvonen et al. 2020). With a population of 109 million, Ethiopia has only 13 eggs per capita per year (Sight and Life 2020). The majority of the eggs originate from backyard production. Consequently, improving egg production could take decades (Shapiro, Gebru, et al. 2017). Addressing these challenges requires increasing egg production and exploring food alternatives that provide similar health benefits. Egg powder, a fully dehydrated form of eggs, presents a viable solution. It is easy to store, has an extended shelf life of 1–2 years, a lower ecological footprint (10%–12%), is easy to transport without refrigeration, has minimal wastage, and carries a lower risk of foodborne illness compared to whole eggs (Hirvonen et al. 2017; Moss et al. 2018). Egg powder is nutritionally identical to whole eggs and more affordable.

Sight and Life received a grant from the Children's Investment Fund Foundation (CIFF) to address the current egg supply limitations. They collaborated with the Ethiopian Public Health Institute (EPHI) to design and conduct a consumer research study to gather insights to help introduce egg powder as an alternative to fresh eggs, rendering it a viable option for lower‐income consumers. The global egg powder market, presently valued at over US$800 million, is projected to reach US$1200 million by 2025 (Schwei et al. 2017). Egg powder, produced through spray‐drying, retains the high nutritional quality of pasteurized whole eggs and provides easier storage, which could help prevent malnutrition in vulnerable populations. This represents a crucial factor for its application in low‐ and middle‐income nations (Pirkwieser et al. 2022). The study is designed to explore potential ways to introduce egg powder in Ethiopia to enhance nutrition, particularly by increasing ASF consumption among young children and women. To our knowledge, there is no published qualitative evidence capturing the experiences and reactions of consumers to egg powder prototypes anywhere in the world. The research addressed the following key questions: (1) How did the egg powder and its prototypes perform in terms of appeal and relevance?; (2) How did the egg powder and its prototypes perform in terms of usage and consumption experience?; and (3) What areas for improvement were identified from a market‐readiness perspective?

## 2 Materials and Methods

A qualitative research methodology was employed to deeply explore the complexities of user reactions, motivations, and feelings toward the new product prototypes. Qualitative methods, such as individual interviews and focus group discussions, allowed participants to provide open‐ended responses, offering the flexibility to uncover new ideas and unexpected insights. This approach enabled the research team to gather rich, detailed data that would likely not have surfaced if more structured, closed‐ended questions had been used.

### 2.1 Study Prototypes

The research design for this study was based on formative research conducted in Addis Ababa, Amhara, Southern Nations, Nationalities and Peoples' Region (SNNPR) and Oromo regions. The study examined current meal practices and helped identify four potential ways to incorporate egg powder. Table 1 demonstrates the different prototypes selected for testing. The four prototypes are: (i) Plain Egg Powder (for consumption as scrambled eggs or firfir); (ii) Shiro (a form of stew made from chickpea flour) with egg powder (referred to as Shiro‐mix); (iii) porridge mixed with egg powder; and (iv) egg powder scrambled with injera (a local flatbread), also known as injera firfir.

**TABLE 1:** Egg powder prototypes that were decided for the consumer research study.

Suggested prototype	Target demographic	Opportunity
1. Plain egg powder	All demographics	Introducing egg powder to the Ethiopian market would also introduce unique values and benefits in relation to accessing eggs, storage. and management, as well as pasteurization and health benefits.
2. Shiro‐mix	All demographics	Shiro emerged as the most widely prepared food in Ethiopian households. But it was also the food that most felt did not have enough nutrients. Introduction of egg powder to Shiro would provide a value proposition to consumers that yields high functional benefits to them.
3. Porridge (to be tested as baby porridge)	6 months–24 months of age infants.	Most mothers were currently adding boiled and mashed egg into the porridge they feed their infants. Therefore, a porridge‐ egg mix would present unique values to the mother and her infant.
4. Injera firfir	All demographics except children under 24 months of age	Injera firfir (a meal made from scrambled injera in a sauce of different choices, mainly onion and tomato‐based sauce) was also commonly prepared when eggs were bought and was enjoyed by the entire household. Mainly, adults and children above 24 months consume injera firfir due to the meals texture which require being chewed somehow.

To ensure a realistic experience for the participants, the four prototypes were developed with careful attention paid to the packaging. Since the research team aimed to introduce new product variants that were not yet available in the market, packaging became the primary means of communicating the product's purpose and nutritional benefits (e.g., ingredient composition, preparation methods, and nutritional information). Therefore, the following elements were incorporated into the package design:

1. **Labels:** Front and back labels were designed to include essential information specific to each prototype.
2. **Nutritional Information:** Nutritional details, tailored to the serving size, were provided for each prototype.
3. **Mix Recommendations:** For prototypes involving mixes (porridge and Shiro mix), the recommended brands to pair with the egg powder were specified.
4. **Instructions:** Basic preparation and cooking instructions were included in a recipe booklet provided to participants.
5. **Measuring Tools:** Measuring cups and teaspoons were supplied to ensure consistent portioning across prototypes.
6. **Translations:** All elements, except brand recommendations, were translated into Amharic and Afan Oromo for accessibility

Ethical approval was received from the Ethiopian Public Health Institute (EPHI) with protocol number EPHI‐IRB‐237‐2020.

### 2.2 Study Design and Sample

Participants were selected using purposive sampling, meaning they were selected because they possessed experience and knowledge aligned with the study with high relevance to the research questions. A total of 38 participants were interviewed. The individual interviews were conducted face to face. Data collection and analysis continued until saturation occurred (i.e., the point at which no new significant themes emerged). Although observations at home and during food shopping were initially planned, they could not be conducted due to the COVID‐19 pandemic.

Participant inclusion criteria included the following: (1) Mothers with at least one child under the age of 59 months, (2) Pregnant women and lactating mothers, (3) Decision makers in the household when it comes to what foods/groceries to buy for their family, (4) Literate, to ensure comprehension of the recipe booklet and label elements, (5) Not averse to the consumption of eggs, (6) No history of egg allergies (including family members), and (7) Belonging to lower‐income categories.

The study locations were chosen to provide a representative sample of regions with high levels of nutritional deficiencies, covering both urban and rural settings for market‐based and humanitarian aid distribution models. The selected locations included: Jimma (Oromiya region), Dessie (Amhara region), Hawassa (SNNPR region), and Addis Ababa (the nation's capital). Table 2 shows the regional nutrition profile of the four regions included in the study.

**TABLE 2:** Distribution of study participants per location and regional profile.

Region	Regional profile		
	Height for age (Stunting)	Weight for height (Wasting)	Weight for age (Indiscriminate)
Addis Ababa	14%	2%	5%
Amhara	41%	8%	27%
Oromia	36%	5%	16%
SNNPR	36%	6%	20%

### 2.3 Procedure

The first part of the procedure involved an in‐home usage test (IHUT) where each participant was provided with the four prototypes and the recipe booklet (examples provided below). Participants were encouraged to test each prototype at least two to three times over 7 days as part of their daily meals and refer to the recipe booklet as much as needed.

After the 7‐day usage period, participants took part in Mini‐Friendship Group sessions. These sessions brought together three to five participants who were familiar with one another and willing to share their experiences collectively. Trained research staff conducted semi‐structured interviews. Each session was audio‐recorded and lasted approximately 1.5 h. Two such groups were held in each region.

While the research team had originally planned to conduct focus group discussions with larger groups, the COVID‐19 pandemic necessitated a shift in methodology. To adapt, we reduced the number of participants per group and ensured they were acquaintances. This rendered them more comfortable discussing their experiences outside their homes.

The audio recordings were de‐identified, transcribed verbatim, and translated from the local languages into English, following IRB protocol.

### 2.4 Topic Guide Design

The topic guide for the mini‐friendship groups explored participants' experiences with each prototype across three dimensions: preparation, cooking, and consumption. Questions focused on their initial reactions, how their experiences aligned with their expectations, and their perceptions toward the prototypes in the market (e.g., packaging cue, price suggestions, and target audience).

### 2.5 Data Analysis

**FIGURE 1:**
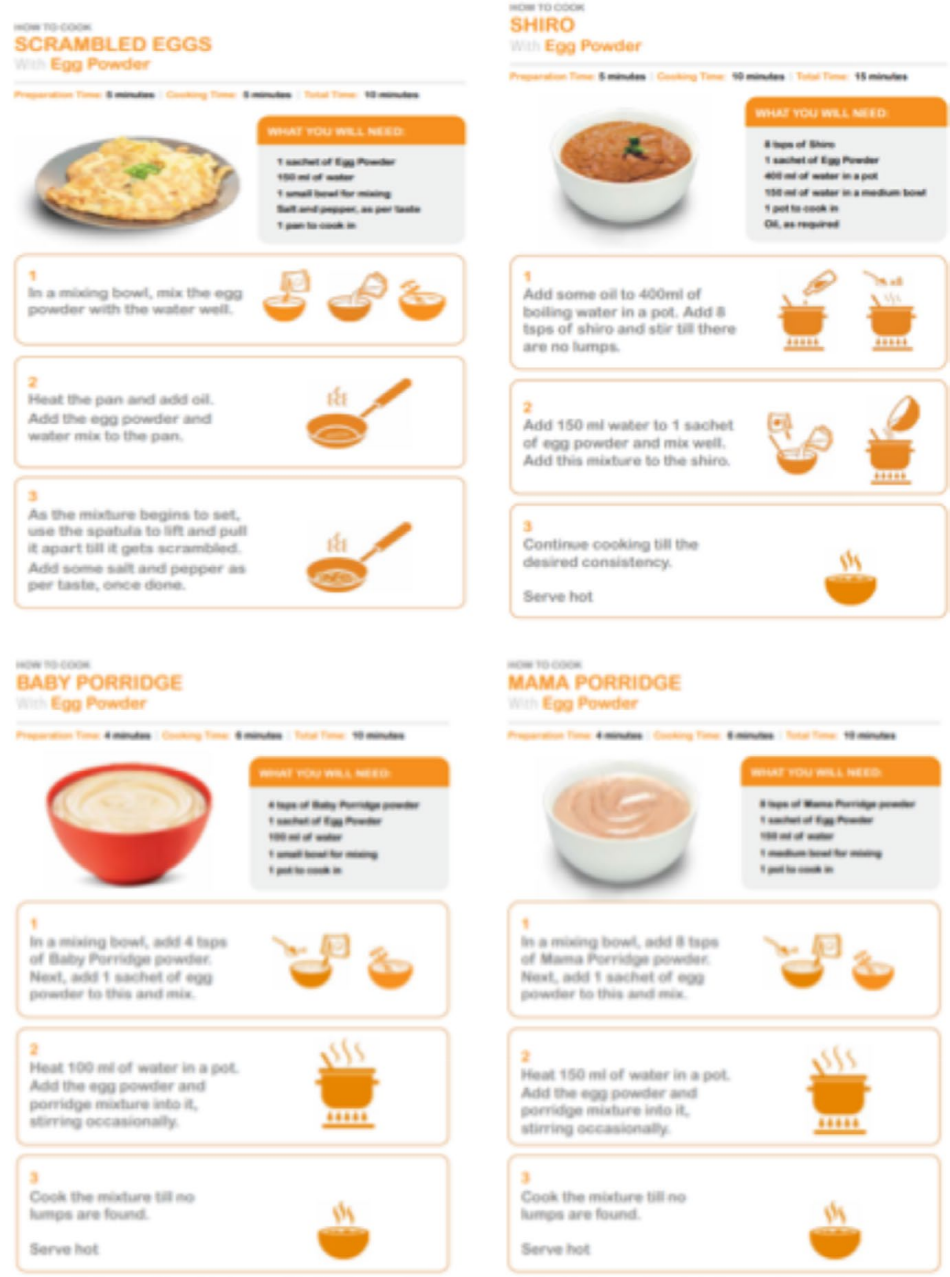
Sample Recipes.

Thematic analysis was employed to analyze the data using the Coffey et al. (1996) method. An inductive approach was used, where transcripts were read and re‐read numerous times by the researcher, to ensure a deep understanding of the data (Imiru 2017). Open coding was followed by developing categories, which were further refined and synthesized to identify broader themes related to each prototype's usage experience. Figure 1 demonstrates sample recipes used.

## 3 Results

The research findings for each prototype are structured in the following way:

1. Perceived benefits of the egg powder product.
2. Feedback on the recipe booklet.
3. Detailed experience during preparation, cooking, and consumption stages.
4. Perceptions of the prototype relevance, pricing, packaging, and place of purchase.

### 3.1 Reactions to the Plain Egg Powder

**FIGURE 2:**
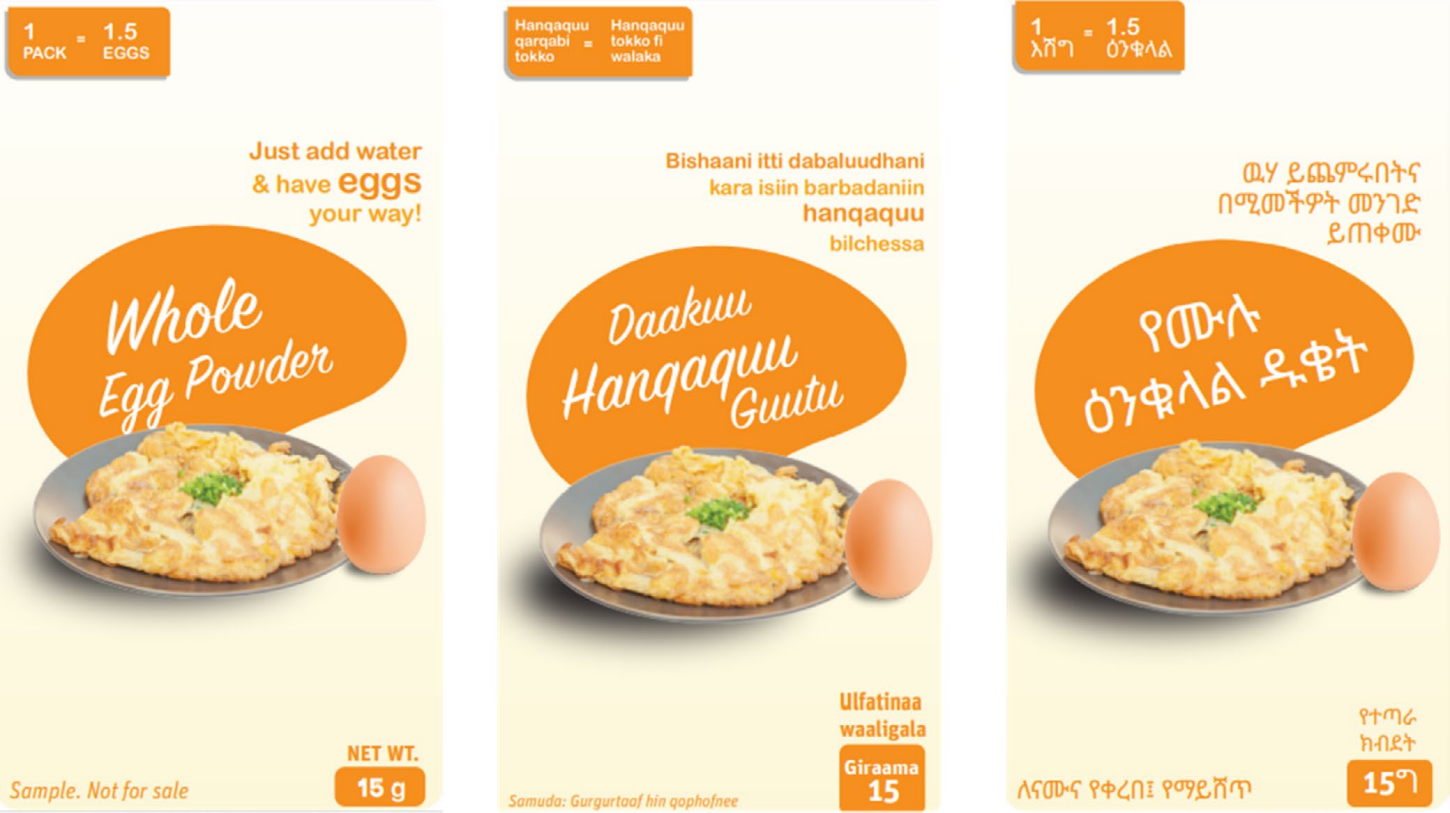
Whole egg powder packaging.

The Plain Egg Powder prototype was placed with all the participants. A picture is available in Figure 2. Table 3 captures the summarized usage experience of the egg powder.

**TABLE 3:** Summary of egg powder usage experience.

PLAIN EGG POWDER	Positive reactions to product usage and experience. The end dish (scrambled eggs) was well liked by all – children, husbands, neighbors, family guests etc. Many participants also added their own twist to the dish—added turmeric/other spices/ meat/potatoes/cabbage etc. Only deterrent was the smell of the egg powder, when mixed with water (before cooking).
From Addis Ababa	*“It was above my expectations. I didn't expect this kind of result when I added water…I didn't expect that it would be beautiful.”*
From Dessie	*“When I cooked it my own way, it has very good taste…I cooked onion, potato, cabbage and spices, then I mixed it with the egg, it changed the taste.”*
From Jimma	*“This one is lighter with a less pleasant smell.”*
From Hawassa	*“I first tested the food for myself. Then, after 30 min I cooked it again and gave it to my 3‐year‐old daughter, she gladly ate it.”*

Overall, the plain egg powder product was well‐received by consumers. Mothers, children, and husbands alike described the final dish as “delicious,” “beautiful,” and “tasty.” Many participants even shared the meal with neighbors, served it to family guests, and offered it to friends.

Perceived benefit of the egg powder: Participants identified several benefits beyond convenience, ease of cooking, and good taste. These included the fact that it is easy to store, can be bought in bulk, and has a longer shelf‐life. Notably, these benefits emerged organically during discussions and were not communicated to participants beforehand. This positive feedback was consistent across all prototypes but is highlighted here specifically for the egg powder.

Feedback on the recipe booklet: The recipe booklet was well received. Participants found the instructions easy to follow, and while the recipe booklet only included a simple scrambled egg recipe with onions, participants reported often adding additional ingredients such as tomatoes, onion, meat, butter, pepper, potatoes, cabbage, turmeric, and other spices. These additions were considered acceptable deviations and demonstrated comfort with the prototype, allowing experimentation and personalization.

Preparation Stage: Preparation was generally considered straightforward, though mixing the egg powder with water took longer than anticipated. Numerous participants felt that less water could be used. Additionally, the smell of the egg powder mixture was described as unpleasant.

Cooking Stage: The recipe booklet suggested a cooking time of 10 min, which did not align with participants' experiences. They recommended either reducing the water amount or increasing the cooking time for better scrambling of the egg powder. Reducing the water quantity was preferred. The unpleasant smell dissipated once cooking began.

Consumption Stage: Everyone who tasted the final product liked it, surpassing expectations. The only negative aspect noted was the appearance of the final dish, which was somewhat pale. Some participants tried to add turmeric, pepper, or berbere to address this.

Prototype Perception: We gauged the perception of each prototype using projective techniques. During the mini‐friendship group discussions, we considered the egg powder a product with aspirational value the appealed to higher‐income consumers as well. They perceived it as modern and innovative, suitable for the entire family, including children over 24 months old. Participants suggested purchasing the product from familiar local stores, neighborhood markets, kiosks, or supermarkets, indicating that, despite its aspirational appeal, it remains accessible. No negative reactions were noted regarding the packaging. All label elements were clear, and the sachet packs were appreciated for easy storage. Some participants suggested adding a dotted line to indicate the tear area on the sachet.

Here, we will also mention the reactions to injera scrambled with egg powder, which all participants were asked to try.

Participants were requested to make Injera Firfir, using the egg powder. It is common practice in Ethiopia for households to have their own injera flour in bulk, or to make their injera flour in bulk. Hence, they only needed to add the egg powder that was provided.

Overall, the reactions to the injera mixed with scrambled eggs made of egg powder were similar to those to the plain. The taste was enjoyed by all, regardless of age.

Preparation Stage: While the recipe booklet instructions were clear, participants preferred to prepare the dish in their own way. The unpleasant smell of the egg powder was noticeable but did not significantly affect the preparation process, which was otherwise familiar.

Cooking Stage: Many participants felt the mixture added beneficial moisture to the injera. Some tried adding the injera to the still‐moist scrambled mixture, allowing for more injera than usual. Deviations from the original recipe were common, showing comfort with the product and willingness to experiment. Participants found the cooking process quick and easy compared to using whole eggs.

Consumption Stage: The final dish received positive reactions for its taste, consistency, and mouthfeel. It was enjoyed by the entire family, and due to its popularity, many participants prepared it multiple times.

### 3.2 Reactions to the Shiro Mixed With Egg Powder

**FIGURE 3:**
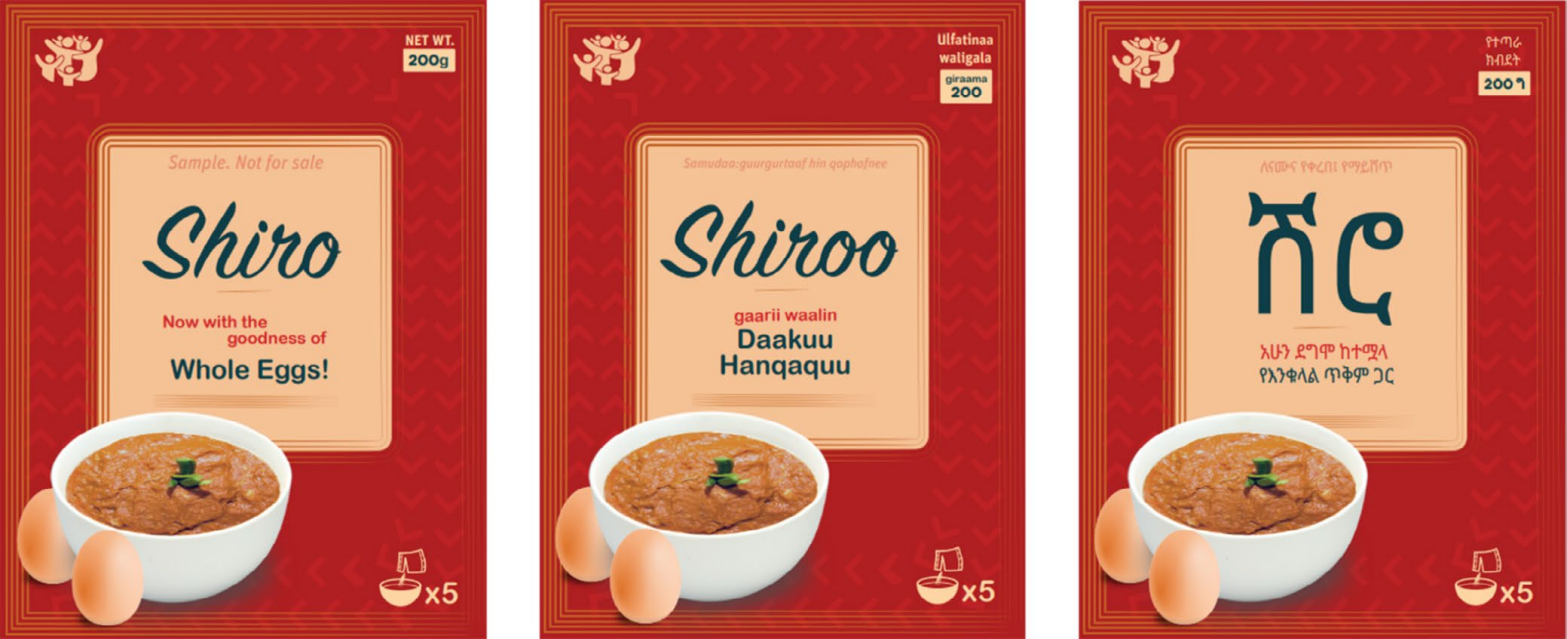
Shiro mixed with egg powder packaging.

The Shiro Mixed with Egg Powder prototype was placed with all the participants. A picture is available in Figure 3. Table 4 captures the summarized usage experience of the Shiro‐mix.

**TABLE 4:** Summary of usage experience for Shiro‐mix.

SHIRO‐MIX	Positive reactions from all participants again Even those who had never tried egg with Shiro, liked the taste. Some did not like the Wow Shiro product, which was placed, so they used the egg powder with their own Shiro at home, and liked it
From Addis Ababa	*“I cooked it for dinner the day I received the product. It was splendid. I enjoyed it very much.”*
From Dessie	*“When I prepared it, it had a good taste but not that much and its color was not good, but I have tried it with my own Shiro from my home then it was very good.”*
From Jimma	*“I will recommend it because my husband loved it.”*
From Hawassa	*“It was beyond my expectation too. I also tried the egg powder with my own Shiro powder too and still it tasted good.”*

Overall, the Shiro‐mix was well‐received by consumers, with mothers, children, and husbands all appreciating its taste and smell. Participants included those who had not previously mixed eggs with Shiro and those familiar with this combination. The Shiro‐mix was highly appreciated by both groups, with many describing it as ‘tastier’ than regular Shiro and noting that the egg bits were not discernable. The dish was enjoyed by the entire family and was even shared with others. It was considered a good one‐meal solution for the whole family, including children over 24 months old. Pregnant women were also seen as a good target since they could consume this without it being too heavy on the stomach.

Regarding smell, the egg powder mixed with ShiroShiro was reported to have a less unpleasant odor, and fewer complaints were noted.

Preparation Stage: The smell of the egg powder did not notably impact participants due to their cooking the Shiro first and then mixing in the egg powder, leading to fewer negative comments.

Cooking Stage: Participants made some minor deviations from the recipe. Some followed the instructions closely, while others adapted the process according to their own preferences. A few respondents expressed dissatisfaction with the Shiro flour provided and preferred to use their own. Additionally, some found the amount of Shiro powder excessive and adjusted it according to their taste. These minor adjustments were made based on individual experiences and preferences.

Consumption Stage: The final product received positive feedback for its taste and consistency. It was described as “tasty,” “luscious,” “delicious,” “smells great,” and “nice color.” The addition of egg powder was noted to enhance the flavor, and the combination was well‐liked by all family members.

Regarding the perception of this prototype, participants felt that it would appeal to all family members rather than a specific demographic. This broad appeal is likely due to Shiro's status as a staple household dish. When discussing where to purchase the product, participants suggested familiar locations such as local stores, neighborhood markets, kiosks, and supermarkets. This indicates that the product is seen as an everyday product that should be easily accessible. No negative feedback was received about the packaging, and all label elements were clear and comprehensible. However, participants suggested that the front label feature Shiro served in a local pot to better reflect traditional associations.

### 3.3 Reactions to Baby Porridge Mixed With Egg Powder

This prototype was tested exclusively with participants who had a child under 59 months old, excluding pregnant and lactating women. For this study, the chosen porridge was the Cerifam: Wheat with Soya variant. Participants were instructed to mix the egg powder into the porridge and prepare it for their child under 59 months old.

**FIGURE 4:**
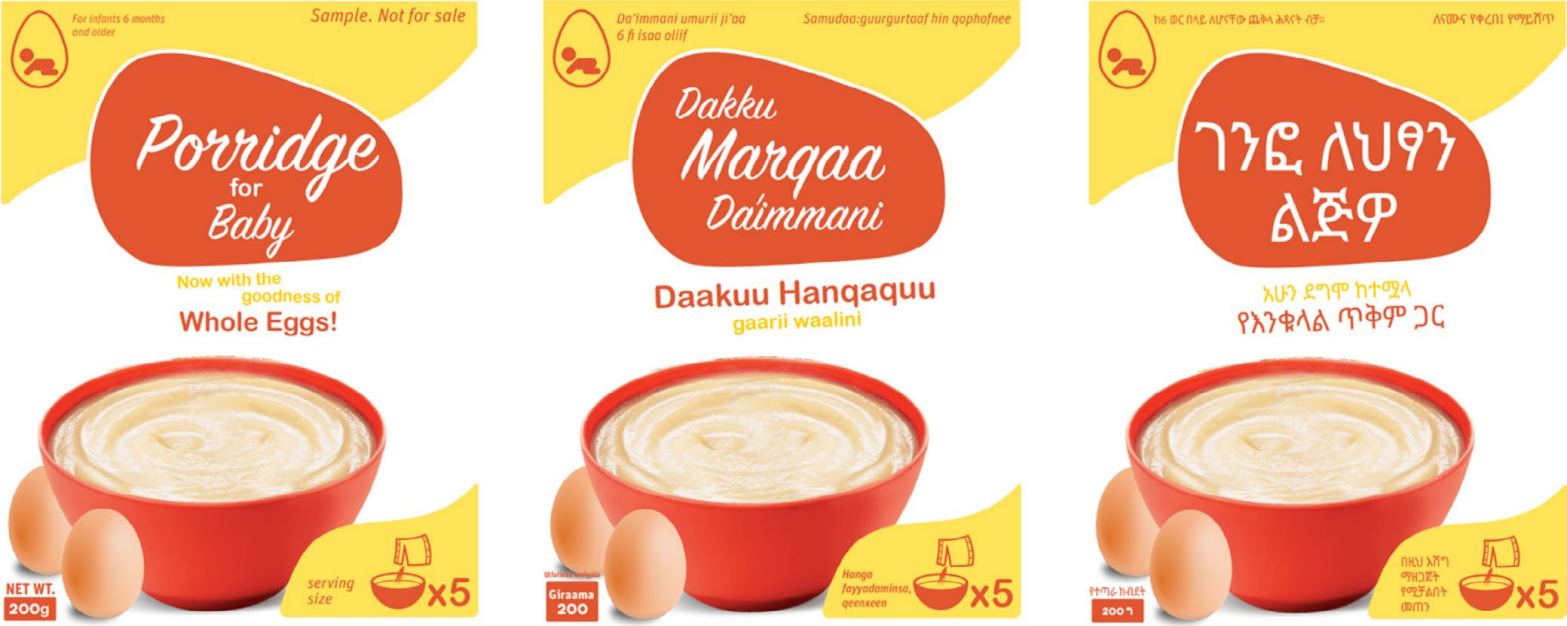
Baby Porridge mixed with egg powder packaging.

A picture of the Baby Porridge mixed with egg powder prototype is available in Figure 4. Table 5 captures the summarized experience of Baby Porridge mixed with egg powder.

**TABLE 5:** Summary of usage experience for baby porridge with egg powder.

BABY PORRIDGE + EGG POWDER	This prototype received largely positive responses across the groups The addition of egg was liked by all the mothers, across the four locations The porridge itself was subject to personal preferences, hence the divided responses were mostly due to the taste of the porridge
From Addis Ababa	*“My kids like Cerifam but when it is with egg they do not like it.”*
From Dessie	*“My children have eaten it and we also taste it at first to know the taste so, it is very good my child has liked it a lot.”*
From Jimma	*“I prepared it based on the recipe… and my daughter didn't eat it because she didn't like sweet taste.”*
From Hawassa	*“My children loved it very much because they normally love eating Cerifam.”*

Overall, the reactions to the baby porridge mixed with egg powder were positive. The taste and mouthfeel of the final porridge dish were well‐received, though there were some negative reactions related to the specific type of porridge used. Reactions varied depending on whether the child was familiar with Cerifam. Children who had never tasted Cerifam before did not enjoy the dish as much as they were unaccustomed to its flavor and texture.

Preparation Stage: The smell of the egg powder mixed with the Cerifam porridge was noted to be less unpleasant, with few complaints reported. The egg powder mixed well with the porridge as well.

Cooking Stage: No significant issues were observed during cooking. The porridge cooked and mixed well with egg powder. The smell and texture of Cerifam predominated in this stage, with respondents noting that the texture was smooth and fine, resembling baby food. The egg powder blended seamlessly into this texture.

Consumption Stage: Reactions to the final dish were mixed. In cases where the porridge was well‐received, children enjoyed it, and the addition of egg did not change the final dish in any perceptible way. The addition of egg powder was also liked by all mothers. Conversely, in cases where it was disliked, the sweet taste of the porridge and the flavor of the added egg were criticized. The ideal consistency was described as ‘stout’ and not ‘fine.’

Perception: Participants viewed this prototype as appealing to all family members and considered it to be modern, innovative, and convenient. The health benefits were not questioned, and adding egg powder was generally welcomed. It was associated with a caring, knowledgeable mother seeking to provide her child with the best nutrition. Participants suggested purchasing the product from local stores, neighborhood markets, kiosks, supermarkets, and pharmacies, indicating a need for trust given the product's target age group. No negative feedback was received regarding the packaging. However, one participant suggested that a front label featuring a child happily eating porridge could attract more attention. Additionally, cardboard packs, like those used by Cerifam, were recommended for better storage.

### 3.4 Willingness to Pay for the Prototypes

**FIGURE 5:**
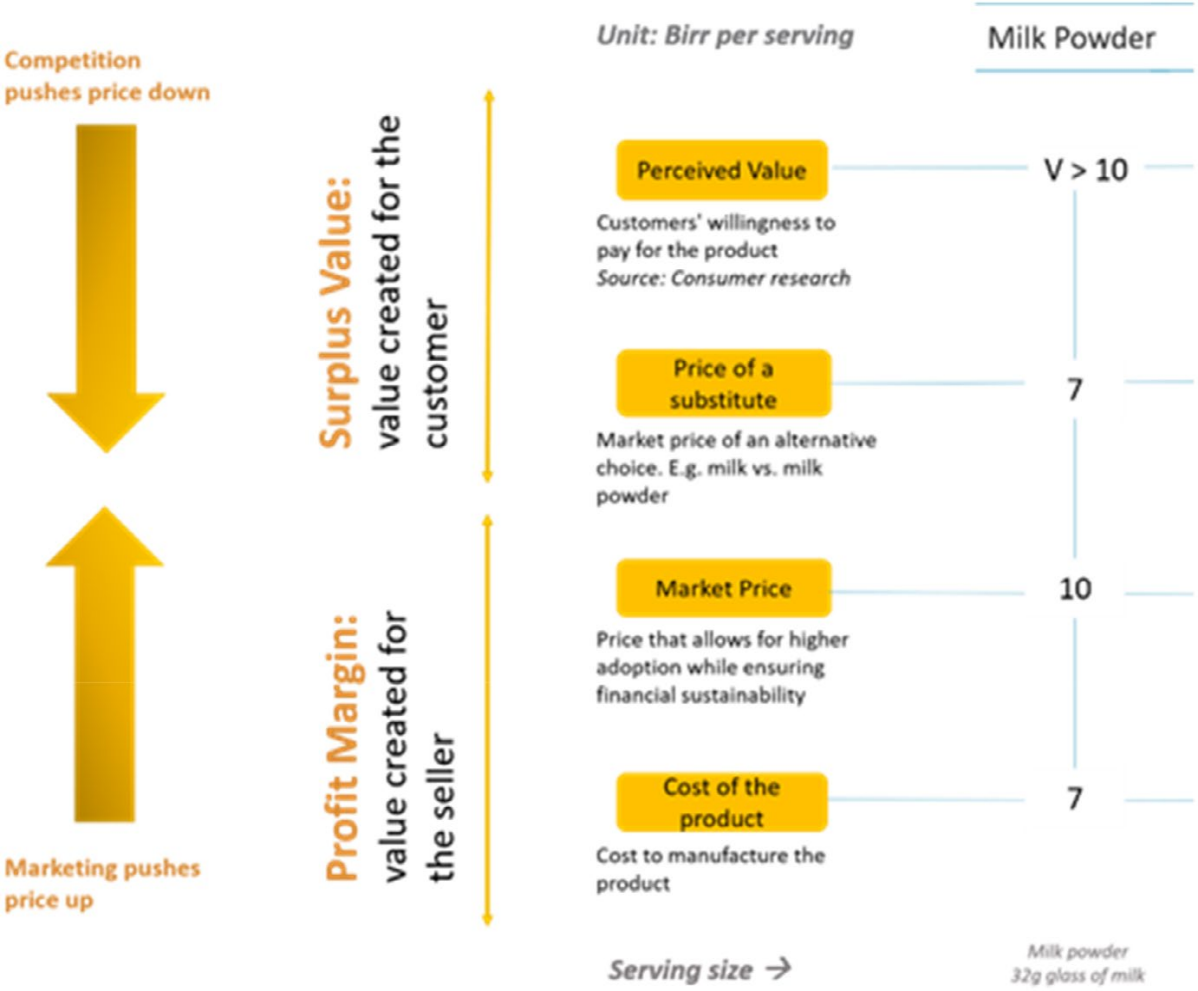
Willingness to pay framework.

The research explored consumers' willingness to pay for three prototypes of egg powder if available in the market. The willingness to pay framework (Figure 5) illustrates the relationship between factors such as: customers' willingness to pay for the product (perceived value), price of an existing market alternative (price of the substitute), the lowest price at which the new product can be sold to maintain financial sustainability (market price) and cost of manufacturing the product (cost of the product).

The framework also introduces two key concepts: (1) Surplus Value, which is the additional value created for the consumer, calculated as the difference between perceived value and the price of the substitute, and (2) Profit Margin, which is the value created for the seller, defined as the difference between market price and cost of the product. Both the surplus value and profit margin are influenced by competition and marketing. Competition from similar products at lower prices can reduce surplus value and profit margins. Conversely, marketing increases the profit margin by focusing on the benefits of the new product and enhancing consumer's willingness to pay.

In the case of the egg powder, consumers compared its value to be the same price as a whole egg at 5 to 6 birr per serving. At 3.5 birr per serving, the manufacturer of egg powder is economically viable. The perceived value (5.0 to 6.0 birr) is higher than the market price (3.5 birr), indicating that consumers might be willing to pay more than the market price (Table 6).

**TABLE 6:** Cost–price analysis for egg powder as a standalone product, egg powder as an ingredient in porridge and Shiro (all prices are in Ethiopian birr).

Format	Egg powder	Porridge	Shiro
Serving size	10.2 g ~ 1 egg	50 g	50 g
Perceived value	5.0 to 6.0 birr	30.0 to 40.0 birr	9.0 birr
Price of a substitute	5.0 (whole egg) birr	19.0 birr (regular porridge)	9.0 birr (regular shiro)
Market price	3.5 birr	18.0 birr	7.0 birr
Cost of the product	2.7 birr	14.0 birr	5.0 birr

Consumers are willing to pay nearly twice as much for porridge with egg powder as an ingredient in comparison to plain porridge. Since porridge is a well‐established consumer category in the country with many different brands, consumers have a higher perceived value for the prototype introducing egg powder as a star ingredient.

In the case of Shiro, the cost of the produce with egg powder is lower than plain Shiro by 4 birr per serving. The manufacturer can be profitable at a market price of 7 birr per serving. Unlike porridge, consumers are willing to pay the same price (9 birr per serving) for Shiro blended with egg powder compared to a blend of a whole egg and Shiro (chickpea stew). This is because Shiro lacks differentiation, is currently unbranded and is mostly produced by the cottage industry. This means consumer perceive all shiro to be the same regardless of who makes it. When Shiro market grows in the future, branding could allow for premium pricing and a cross‐subsidy strategy.

Overall, the data suggests that consumers perceive higher value in all three products compared to their current market prices, indicating a willingness to pay more. This is a positive sign for potential price adjustments to increase profitability while remaining competitive against substitutes.

## 4 Discussion

The in‐home usage of egg powder elicited positive reactions, particularly regarding sensory aspects, though clear areas of improvement were identified. The Shiro and baby porridge prototypes were particularly well‐received, while the Mama porridge prototype revealed a preference among adults for savory, less sweet, and textured porridges. Respondents generally expressed confidence in using the products and found them relevant to their needs.

Beyond nutrition and taste, the egg powder was appreciated for its practical advances in the kitchen. Consumers valued its easy storage, long shelf life, durability, and convenience. These factors align with other consumer research findings in Ethiopia, where convenience in preparation and access significantly influences food choices and purchase intentions (Melesse et al. 2019; Karanja et al. 2022).

The reactions to the packaging provide key insights. First, the benefits of the egg powder, including nutritional value, were clearly noted. Consumers recognized the nutritional advantages of the egg powder unaided, highlighting the importance of clear nutritional information on packaging. Such details are critical for influencing purchasing decisions (Imiru 2017). Second, the egg powder is perceived as modern, relevant, and innovative. It carries aspirational value and does not alienate the intended user despite being a completely new product, and it offers functional, emotional and image–related benefits. The packaging's impact—through its innovation, clear information, and appealing design—aligns with the expectations of urban Ethiopian consumers who value packaging that attracts attention and conveys product value (Imiru 2017).

The Government of Ethiopia has expressed a high level of political commitment to improving maternal and child malnutrition, but a lack of innovative intervention hindered the improvement. Eggs remain out of reach for a large segment of the Ethiopian population due to costs and accessibility‐related issues, especially in rural areas. Egg powder, a solution produced in countries where eggs are surplus and hence affordable to LMIC, offers a promising solution to improve nutrition outcomes while offering numerous benefits above and beyond whole eggs. Egg powder can potentially follow the milk powder journey in the Ethiopian context. For instance, powdered milk's share in the budget has increased. In contrast, at the country level, the value of powdered milk imports increased rapidly, amounting to almost 20 million USD in 2015 from just over 5 million USD in 2005 (Minten et al. 2020).

One limitation of this study is the sample size, only 38 participants in total were interviewed, which is not representative of the population but was not intended to be representative as the authors intended to gather information that could be used to inform broader findings regarding the prototypes and a new product development lifecycle. However, previous research has recommended that qualitative studies require a minimum sample size of at least 12 to reach data saturation. Therefore, a sample size of 38 is deemed sufficient for the purpose and findings of this study as data saturation was achieved (Baye et al. 2021).

Perceived values of the products were also explored and collected from this consumer research. Consumer research showed that consumers were willing to pay for egg powder the same as the cost of an egg, which indicates that there is an opportunity to define the value of the product. For example, milk powder in Ethiopia is manufactured and imported for 7 birr per serving (cost of the product). However, customers are willing to pay more than 10 birr (perceived value) because the quality of liquid milk (substitute) in Ethiopia is poor. Hence, consumers are willing to pay at least 3 birr in surplus value for the hygienic and quality standards of milk powder.

A previous analysis of household consumption and expenditure across nine regions and two administrative cities shows that only 0.2% of the total consumption expenditure was allocated for eggs, far below the 2.2% and 4.3% required to allow the consumption of one egg a day by the average and the poorest households, respectively (Baye et al. 2021). Given that egg powder has a willingness to pay comparable to that of an egg, while potentially lowering how much consumers have to pay for the cost of the minimum‐cost nutritious diet (one study shows by an average of 16 percentage points) (Ameye, Bachewe, and Minten 2021) egg powder can have a promising future. This is especially so in the context of high and increasing prices of nutritious foods (Ameye, Bachewe, and Minten 2021).

Overall, egg powder holds significant promise in improving nutritional outcomes in Ethiopia, offering a viable solution to dietary challenges while aligning with consumer preferences and market dynamics.

## Author Contributions

**Rebecca Olson:** writing – review and editing (lead). **Puja Peyden Tshering:** conceptualization (equal), writing – original draft (equal), writing – review and editing (equal). **Kalpana Beesabathuni:** project administration (equal), supervision (equal). **Srujith Lingala:** writing – review and editing (supporting). **Afomiya Mekonnen:** writing – review and editing (supporting). **Masresha Tessema:** writing – review and editing (supporting). **Alemnesh Petros:** writing – review and editing (supporting). **Tarik Taye Birhanu:** writing – review and editing (supporting). **Abeba Ayele:** writing – review and editing (supporting). **Sufia Askari:** writing – review and editing (supporting). **Rowena K. Merritt:** writing – review and editing (equal).

## Ethics Statement

Ethical approval was sought from the Ethiopian Public Health Institute (EPHI) IRB Committee with protocol number: EPHI‐IRB‐237‐2020.

## Consent

Informed consent was obtained from all subjects involved in the study.

## Conflicts of Interest

The authors declare no conflicts of interest.

## Data Availability

The data that support the findings of this study are available on request from the corresponding author. The data are not publicly available due to privacy or ethical restrictions.
